# Effects of deep inspiration breath hold on prone photon or proton irradiation of breast and regional lymph nodes

**DOI:** 10.1038/s41598-021-85401-4

**Published:** 2021-03-16

**Authors:** Bruno Speleers, Max Schoepen, Francesca Belosi, Vincent Vakaet, Wilfried De Neve, Pieter Deseyne, Leen Paelinck, Tom Vercauteren, Michael J. Parkes, Tony Lomax, Annick Van Greveling, Alessandra Bolsi, Damien C. Weber, Liv Veldeman, Werner De Gersem

**Affiliations:** 1 Department of Human Structure and Repair, Faculty of Medicine and Health Sciences, Ghent University, Radiotherapiepark, Corneel Heymanslaan 10, 9000 Ghent, Belgium; 2 Paul Scherrer Institut, Villigen, Switzerland; 3 Department of Radiation Oncology, University Hospital Ghent, Ghent, Belgium; 4 Department of Industrial Systems Engineering and Product Design, Faculty of Engineering and Architecture, Ghent University, Ghent, Belgium; 5 Academic Medical Centre (AMC), University of Amsterdam, Amsterdam, the Netherlands; 6 Radiation Oncology Department, University Hospital of Bern, Bern, Switzerland; 7 Radiation Oncology Department, University Hospital of Zurich, Zurich, Switzerland

**Keywords:** Breast cancer, Radiotherapy

## Abstract

We report on a comparative dosimetrical study between deep inspiration breath hold (DIBH) and shallow breathing (SB) in prone crawl position for photon and proton radiotherapy of whole breast (WB) and locoregional lymph node regions, including the internal mammary chain (LN_MI). We investigate the dosimetrical effects of DIBH in prone crawl position on organs-at-risk for both photon and proton plans. For each modality, we further estimate the effects of lung and heart doses on the mortality risks of different risk profiles of patients. Thirty-one patients with invasive carcinoma of the left breast and pathologically confirmed positive lymph node status were included in this study. DIBH significantly decreased dose to heart for photon and proton radiotherapy. DIBH also decreased lung doses for photons, while increased lung doses were observed using protons because the retracting heart is displaced by low-density lung tissue. For other organs-at-risk, DIBH resulted in significant dose reductions using photons while minor differences in dose deposition between DIBH and SB were observed using protons. In patients with high risks for cardiac and lung cancer mortality, average thirty-year mortality rates from radiotherapy-related cardiac injury and lung cancer were estimated at 3.12% (photon DIBH), 4.03% (photon SB), 1.80% (proton DIBH) and 1.66% (proton SB). The radiation-related mortality risk could not outweigh the ~ 8% disease-specific survival benefit of WB + LN_MI radiotherapy in any of the assessed treatments.

## Introduction

Radiation therapy (RT) after breast-conserving surgery in locally advanced stage breast cancer improves locoregional control and survival^1^. However, the benefit occurs at the expense of acute and late toxicity to the treated region, including but not limited to cardiac events, lung cancers and cancers in the contralateral breast^2–6^. Cardiac injury and cancer induction lead to excess mortality and are dose dependent^7^. RT in prone position allows for dose reductions to lung and heart, hence lowering the risks of radiation-induced cardiac toxicity and lung cancer^8–11^. Furthermore, prone positioning may be advantageous not only in whole breast (WB) irradiation, but also when combined with lymph node (LN) irradiation including the internal mammary (MI) chain^12,13^. However, patient support devices for prone radiotherapy share several drawbacks for locoregional radiotherapy, such as patient discomfort and low set-up precision in the lateral direction^14^. To address these problems, we have previously studied a novel ‘front crawl’ prone position for patients requiring locoregional treatment at Ghent University^12–14^. Accordingly, we have also developed a prone support device (Prone Crawl Breast Couch) to facilitate patient positioning, CT-simulation and treatment. Using the Prone Crawl Breast Couch for the locoregional treatment, without or with inclusion of the MI chain, we showed that dose reductions to lung, heart and other OARs could be obtained beyond what is achievable using advanced supine photon irradiation techniques^12,13^.

The dose to the heart can be further reduced by using deep inspiration breath hold (DIBH) in prone or supine position, rather than with shallow breathing (SB)^10^. Imaging studies in prone position confirm that DIBH retracts the MI chain away from the heart^15^. In a direct comparison of 4 techniques (prone or supine, SB or DIBH), prone DIBH achieved the lowest heart and lung doses for left-sided whole breast (WB) treatments^10^.

In this study, we investigated the dosimetrical effect of DIBH in prone crawl position on heart, lungs and other organs-at-risk (OARs) in both photon and proton plans, for the treatment of WB and LN (including the MI chain) as compared to SB. Afterwards, the mortality risk was compared, from radiation dose-related injury to heart and induction of lung cancer, to the expected survival benefit of WB + LN radiotherapy in this patient population.

## Materials and methods

### Patient selection

Thirty-one patients with invasive carcinoma of the left breast and pathologically confirmed positive lymph node status were included in this study, which was approved by the local ethics board of Ghent University Hospital. The age of the patients, at the time of their CT-simulation, ranged between 29 and 74 years, with a median of 54. All research was performed in accordance with applicable guidelines and regulations and informed consent was obtained from all participants.

### Patient set-up

**Figure 1:**
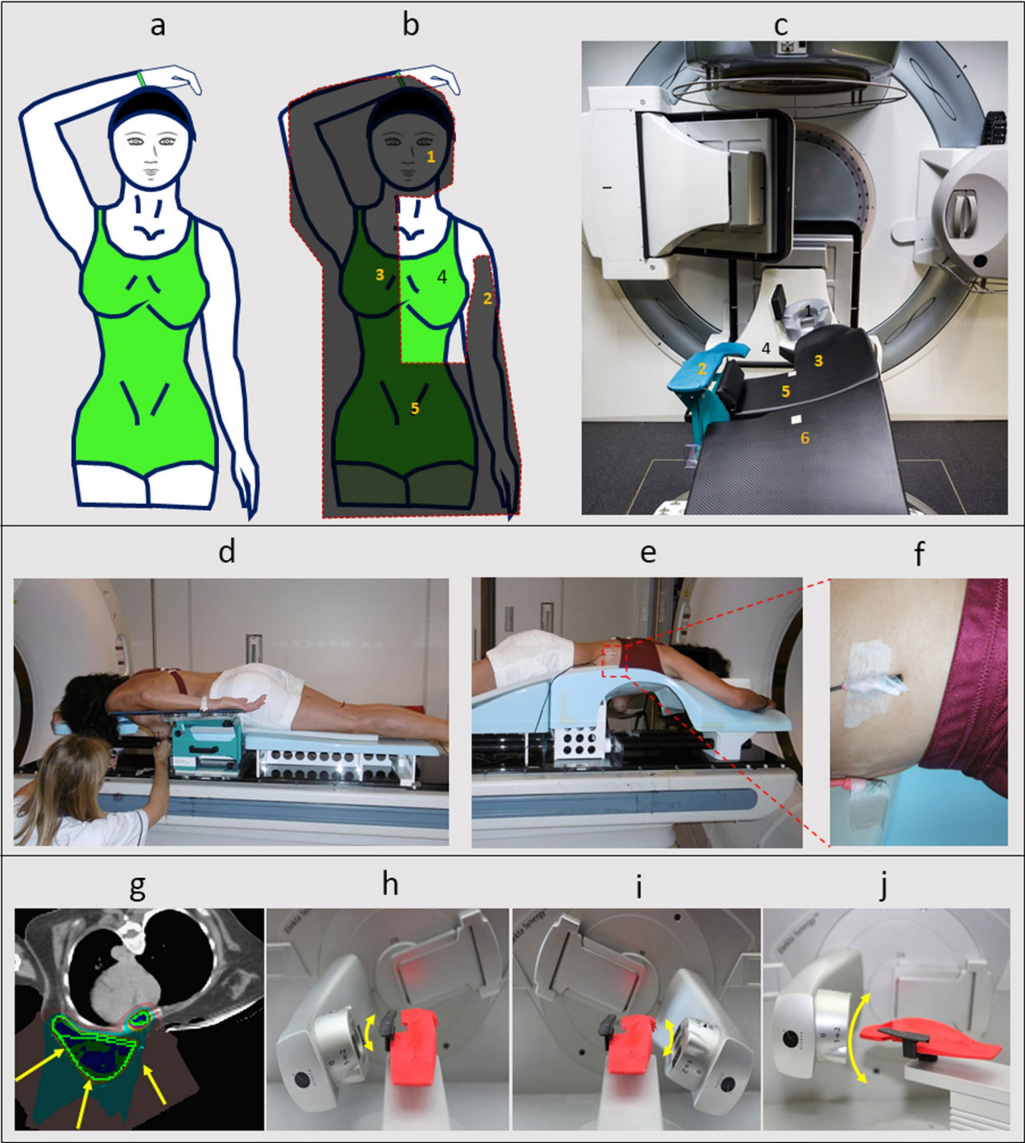
Patient set-up and treatment techniques. **(a)** Illustration of the prone crawl position for irradiation at the left side. **(b)** Projection of the support surface of the Prone Crawl Breast Couch. **(c)** Photograph from above and behind of a Prone Crawl Breast Couch for irradiation at the left side installed on the couch of a linear accelerator. (1. Prone head rest; 2. Arm support blade; 3. Contralateral (untreated) breast support surface; 4. Aperture exposing the ipsilateral (treated) breast and its regional lymph nodes; 5. Lower body support surface and 6. Leg support surface). **(d,e)** Photograph from the ipsilateral **(d)** and contralateral **(e)** sides of a patient positioned for CT-simulation. The ipsilateral breast, lateral thoracic wall and shoulder hardly move with respiration. Therefore, the Respisens sensor system, that monitors the breathing cycle and the DIBH maneuver by real-time registration of the distance between 2 sensors, is placed at the contralateral thoracic wall. **(f)** Close-up of the sensors. The upper and lower sensors are taped to the lateral thoracic wall of the patient and to an immobile part of the Prone Crawl Breast Couch, respectively. **(g)** CT-slice in the transverse plane through the mid-breast with arrows indicating the 3 beam directions used in proton therapy plans. **(h–j)** Model of an Elekta Synergy linear accelerator with scaled 3D-printed Prone Crawl Breast Couch for left side treatment. The arrows give an impression of the arc directions used for photon therapy of the breast and regional lymph nodes. **(h)** Short arc from the ipsilateral side using 0° couch isocenter rotation. **(i)** Contralateral short arc using 0°couch isocenter rotation. **(j)** Para-sagittal arc using 70° couch isocenter rotation.

Patients were positioned on the Prone Crawl Breast Couch (Fig. 1 panels a–d) as described previously^13^. DIBH was monitored using Respisens magnetic sensors (Nomics, Angleur, Belgium) placed on the surface of the Breast Couch and lateral thoracic wall^16^ (Fig. 1 panels e–f). Patients underwent two computed tomography (CT) scans for radiotherapy planning, first in a short DIBH of around 15 s and later in SB, as described previously^17^. Patients were instructed to practice the DIBH maneuver in advance at home. CT-images of 5 mm slice thickness were acquired, starting at below the mandible, and caudally ending below the diaphragm. Neither patient positioning nor scan range were altered between DIBH and SB. This was to assure that the DICOM coordinate system, indicated by the frame of reference UID of the different scans, remained identical.

### Target & OAR definition

Target and OARs delineation and margin generation were performed on a Pinnacle 9.8 treatment planning system (Philips Healthcare, Fitchburg, Wisconsin, USA) as described previously^13^. In brief, the whole breast was delineated up to 5 mm from the skin surface as CTV_WBI. CTV_PC included axillary level II-IV lymph nodes, delineated using the PROCAB guidelines^18^. CTV_MI included the ipsilateral MI lymph nodes. Planning target volumes were obtained by performing a 3 mm isotropic expansion of CTV_PC and a 1 mm isotropic expansion of CTV_MI, thereby creating PTV_PC and PTV_MI, respectively. PTV_WBI was created using a 5 mm margin except towards the skin surface to minimize build-up effects. Photon plan optimization structures were created to reduce influence of dose buildup underneath the skin on plan optimization and to account for breast swelling. The whole heart was delineated in accordance with guidelines proposed by Feng et al.^19^. The left anterior descending artery (LAD) and the apex were individually delineated. Left and right lungs were contoured separately using the automatic segmentation by Hounsfield unit options provided in Pinnacle 9.8 with threshold 800–4096. Contralateral breast was delineated up to the skin. Thyroid was delineated where visible. The esophagus was delineated starting cranially from the inferior margin of the cricoid and ending inferiorly at the gastro-esophageal junction.

### Treatment planning

For proton plan optimization, planning CT and structures acquired and contoured at Ghent University Hospital were imported in the Paul Scherrer Institut (PSI) treatment planning system in Switzerland. The pencil beam scanning (PBS) proton plans were computed using PSIplan, an in-house treatment planning system^20^. For each proton plan, three oblique anterior fields were used with about 30° of angular spread between them (Fig. 1 panel G). Combined target optimization of spot weights was done simultaneously for all fields using intensity modulated proton therapy (IMPT)^21,22^. Detailed information for proton plan optimization has been described previously by Speleers et al.^13^.

For photon plan optimization, a non-coplanar multiple overlying short arc VMAT technique was used, which exploits optimal beam directions and reduces low-dose spread to the OARs^13^ (Fig. 1 panels H–J). VMAT planning tools, developed at Ghent University Hospital as extensions of GRATIS treatment planning platform (Sherouse systems, Inc., Chapel Hill, NC, USA), are described elsewhere^19^. The final dose calculation was performed using the convolution-superposition dose computation engine in Pinnacle 9.8.The objective was a median dose of 40.05 Gy/GyRBE (prescribed dose) in 15 fractions to the optimization structures related to PTV_WBI, PTV_PC and PTV_MI, with 95% of the volumes covered by ≥ 95% of the prescribed dose and no more than 5% receiving 105% of the prescribed dose. Dose per fraction to the breast, the level II-IV axillary and the ipsilateral MI lymph node regions was 2.67 Gy for photons or 2.67 GyRBE/fraction for protons..

### Dose statistics

Dose statistics are referred to as D*n* (the minimal absolute dose delivered to *n* % of the volume) or V*n* (the volume percentage receiving ≥ *n* Gy/GyRBE). D_02_ and D_98_ were used as surrogates for maximum and minimum dose, respectively. Dose is reported for PTV_WBI, PTV_MI and PTV_PC. The dose homogeneity index was defined as (D_02_–D_98_)/D_mean_. For statistical comparison, two-tailed paired t-tests were performed and p ≤ 0.05 considered statistical significance.

Mean dose to heart, LAD, apex, lungs (both lungs together), ipsilateral and contralateral lung, thyroid, esophagus, brachial plexus, spinal cord and contralateral breast are reported.

The thirty-year mortality risk from radiation-induced cardiac injury and lung cancer for a reference patient, a 50-year old woman at the time of irradiation, was calculated from mean heart dose and mean dose to both lungs according to Taylor et al.^7^. Risk rates of 0.075%/Gy and 0.3%/Gy were used for patients without or with cardiac risk factors, respectively, to calculate cumulative 30-year risk of dying from radiation-induced heart disease^7^. Risk rates of 0.06%/Gy and 0.88%/Gy were used for non-smoking or smoking patients, respectively, to calculate cumulative 30-year risk of dying from radiation-induced lung cancer^7^. Cumulative risk of dying from heart disease and/or lung cancer was calculated as 1 − (1 − P_h_)(1 − P_l_) where P_h_ and P_l_ are the risks to die from radiation induced heart disease or lung cancer, respectively. We then made a risk–benefit classification for different risk categories of cardiac events and lung cancer. The line of regret^13^ is calculated for the 8% disease-specific absolute 30-year survival benefit of radiotherapy, based on literature^23^. For each patient, mean heart and lung doses are plotted onto this graph, showing whether the benefits of radiotherapy can (below the line of regret) or cannot (above the line of regret) outweigh the added risks of radiotherapy. The equation of the 8% line of regret (Fig. 4) is based on Taylor’s data^7^ where 0.92 is derived from the 8% (1–0.08 = 0.92), 0.003 stands for the risk/Gy(RBE) for cardiac death for high-risk patients and 0.088 stands for the risk/Gy for lung cancer death for high-risk patients.

## Results

### Dose to target structures

The dose homogeneity index (HI) was 13.1% for photon DIBH, 12.8% for photon SB, 8.80% for proton DIBH and 9.38% for proton SB. HI-differences between photon and proton plans are significant for SB (p = 0.002) and DIBH (p = 0.005). Average left breast volume was not significantly different at 398 cm^3^ (range: 45–921) and 393 cm^3^ (range: 53–902) in SB and DIBH, respectively. Dose objectives were met for all targets in all plans.

**Figure 2:**
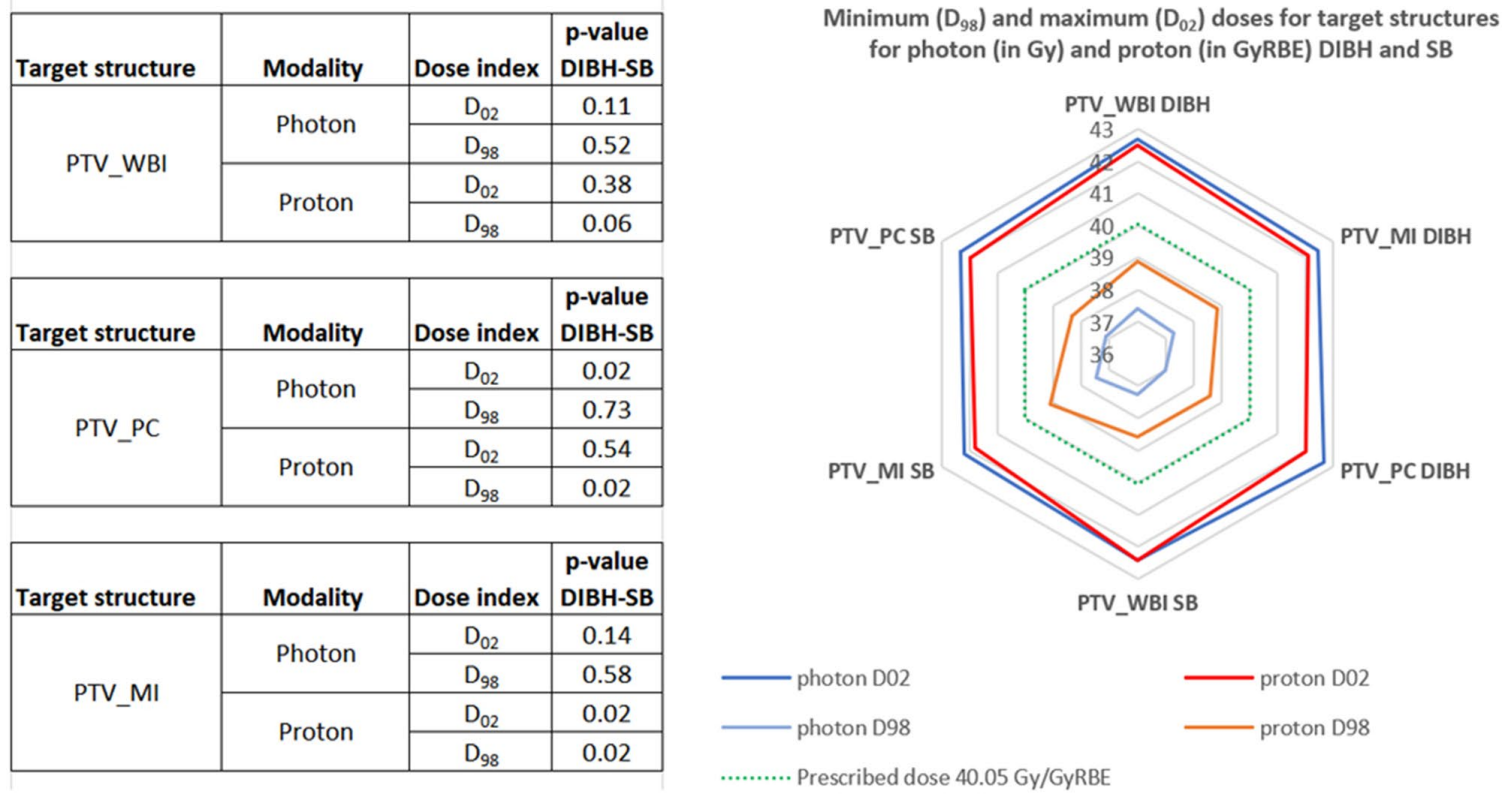
Dose indices of target structures for photon and proton plans. In the inserted table the parameter ‘p-value DIBH-SB’ is the p-value of a two-tailed t-test for comparing the dose values between DIBH and SB, where p ≤ 0.05 is considered statistical significance.

Figure 2 shows minimum (D_98_) and maximum (D_02_) dose indices for target volumes in DIBH and SB for both photon and proton plans. For photon plans, there were no significant differences in D_98_ between DIBH and SB for all target structures. For proton plans, there were no significant differences in D_98_ between DIBH and SB for PTV_WBI. The average D_98_ in proton plans was 0.25 GyRBE higher in DIBH than in SB (p = 0.02) for PTV_PC and 0.27 GyRBE lower in DIBH than in SB for PTV_MI (p = 0.02).

Regarding the D_02_ for PTV_WBI, there were no significant differences between photon DIBH and SB (p = 0.11) and between proton DIBH and SB (p = 0.37). For PTV_PC, the average D_02_ was 0.36 Gy higher in photon DIBH than in photon SB (p = 0.02) and no significant difference was found between proton DIBH and proton SB (p = 0.53). For the average maximum doses of PTV_MI, there was no significant difference between photon DIBH and SB (p = 0.14), but the average maximum dose was 0.29 GyRBE higher in proton DIBH than in proton SB (p = 0.02).

### Dose to organs at risk

**Figure 3:**
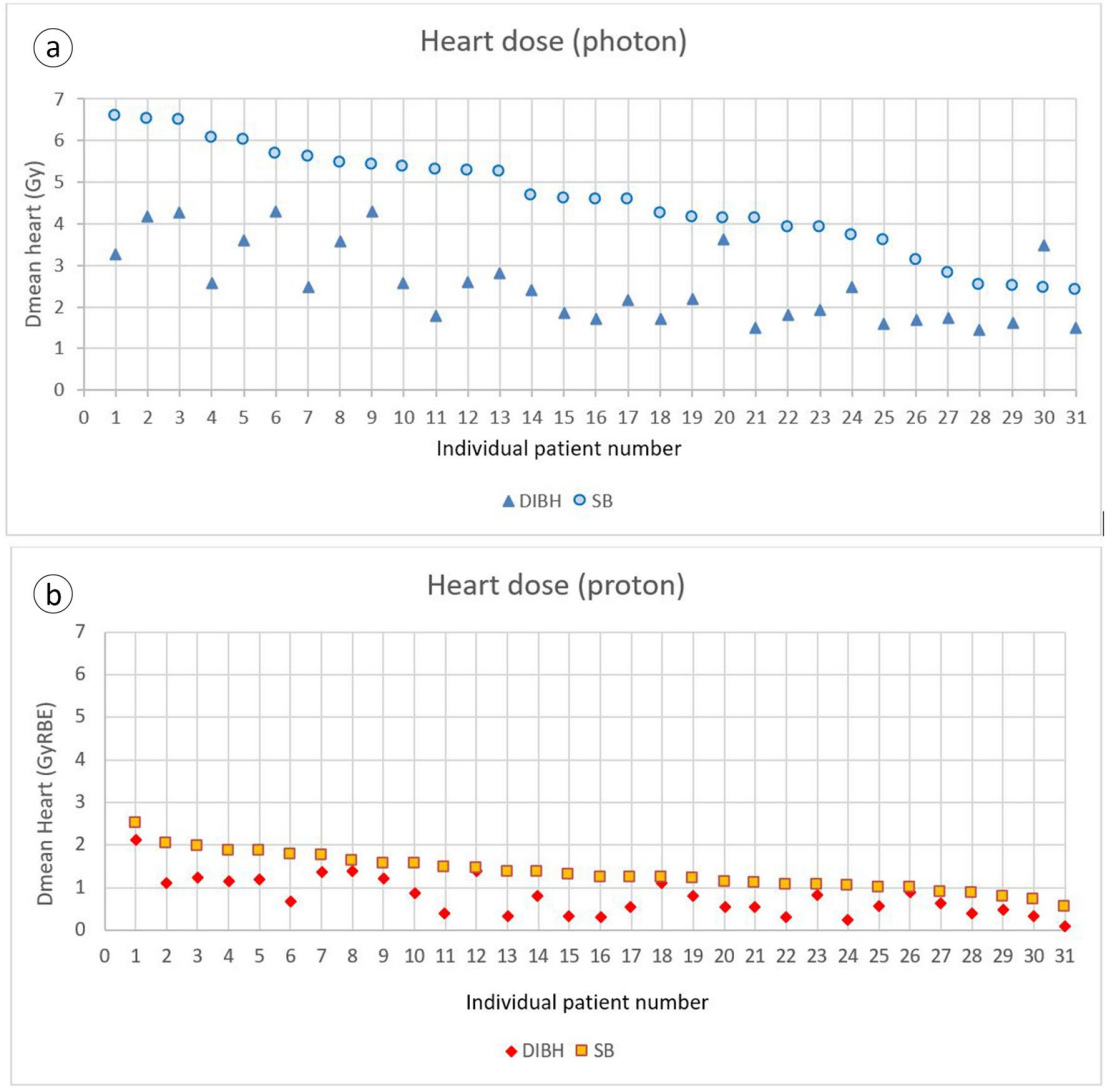
Plots of individual mean heart dose ranked according to decreasing SB dose for **(a)** photons and **(b)** protons.

Dose indices of OARs are summarized in Table 1. The DIBH-technique, compared to SB, significantly decreased mean dose to heart in both photon and proton plans. Figure 3 provides an overview of the individual mean heart doses for photon (upper panel) and proton (lower panel) irradiation, ranked according to decreasing mean SB-dose. The mean heart dose reduction in DIBH, compared to SB, for photon and proton was on average 2.0 Gy (range: − 1.0 to 3.5) and 0.56 GyRBE (range: 0.1–1.1), respectively.

**Table 1:** Dose indices of organs-at-risk.

Dose (Gy /GyRBE)	Photon					Proton					Ph/Pr DIBH	Ph/Pr SB
	DIBH		SB		p-value	DIBH		SB		p-value	p-value	p-value
Heart (mean)	2.54	(1.43–4.31)	4.55	(2.40–6.58)	< 0.001	0.78	(0.10–2.11)	1.34	(0.53–2.50)	< 0.001	< 0.001	< 0.001
Heart apex (mean)	4.15	(1.24–14.05)	11.07	(1.51–26.33)	< 0.001	3.44	(0.04–9.56)	4.35	(0.02–12.00)	0.17	0.05	< 0.001
LAD (mean)	6.50	(2.04–17.56)	11.09	(3.02–23.16)	< 0.001	2.19	(0.02–7.56)	2.26	(0.02–14.64)	0.90	< 0.001	< 0.001
Lung left (mean)	4.72	(3.38–6.85)	5.44	(3.93–7.43)	< 0.001	3.70	(2.26–5.57)	3.05	(1.74–4.68)	< 0.001	< 0.001	< 0.001
Lung right (mean)	0.93	(0.26–2.90)	1.04	(0.23–3.36)	0.17	0.14	(0.03–0.46)	0.08	(0.03–0.36)	0.002	< 0.001	< 0.001
Lungs (mean)	2.71	(1.72–4.45)	3.08	(2.04–4.89)	< 0.001	1.78	(1.05–2.80)	1.44	(0.79–2.34)	< 0.001	< 0.001	< 0.001
Esophagus (mean)	2.97	(1.09–6.35)	3.38	(1.16–6.91)	0.02	2.99	(0.24–6.95)	3.27	(0.06–7.43)	0.14	0.90	0.66
Esophagus (D_02_)	20.67	(5.17–39.23)	21.23	(2.97–40.35)	0.67	25.45	(3.44–40.98)	25.20	(0.54–40.51)	0.81	0.001	0.02
Thyroid (mean)	5.08	(1.43–16.37)	5.22	(0.78–11.90)	0.77	8.52	(0.77–18.48)	8.49	(0.58–20.26)	0.96	< 0.001	< 0.001
Brachial Plexus (D_02_)	43.83	(40.16–49.85)	42.85	(37.36–(47.82)	0.01	41.16	(40.12–42.16)	41.05	(38.32–42.33)	0.40	< 0.001	< 0.001
Spinal cord (D_02_)	4.15	(1.06–16.60)	4.38	(0.99–18.89)	0.69	0.19	(0.04–2.67)	0.076152	(0.04–0.60)	0.19	< 0.001	< 0.001
Spinal cord (isotropic expansion 5 mm (D_02_)	5.50	(1.13–18.10)	5.90	(1.24–25.31)	0.53	0.29	(0.04–3.01)	0.141765	(0.04–0.88)	0.11	< 0.001	< 0.001
Contralateral breast (mean)	1.15	(0.25–2.57)	1.18	(0.46–1.88)	0.59	0.11	(0.02–0.25)	0.09	(0.02–0.25)	0.04	< 0.001	< 0.001

Average values (range) for 31 patients. P-values for paired t-test. Column 6: p-values for photon DIBH versus photon SB plans. Column 11: p-values for proton DIBH versus proton SB plans. Column 12: p-values for DIBH photon versus DIBH proton plans. Column 13: p-values for SB photon versus SB proton plans.

As seen in Table 1, DIBH also resulted in a significantly lower mean dose for the esophagus for photons, but not for protons. On average in photon plans, left lung mean dose decreases about 13% by using DIBH, whereas in proton DIBH plans the average mean left lung dose increases by about 21%. No significant difference was observed both for proton and photon on the mean dose to the contralateral breast.

As for relevant maximum doses, we saw significantly higher doses in photon than in proton plans for brachial plexus and spinal cord. However, for the maximum dose on the esophagus and the mean thyroid dose, proton plans show significantly higher doses in both breathing settings.

### Analysis of regret

Thirty-year risk estimations of dying from radiation induced heart disease or lung cancer (for a 50-year old reference patient) are given in Table 2. Here, two different characteristics have been taken into account. The first distinction is based on the presence of cardiac risk factors. The second distinction is based on the presence of lung cancer risk factors, in this case long-term smoking behaviour. These rates, multiplied with the average mean heart or lung dose in Gy or GyRBE, give an indication of the radiation-induced cardiac or lung cancer mortality risk, respectively, for the different groups.

**Table 2:** Risk estimations for radiation-induced mortality.

WBI + LNI + MI	Photon					Proton					Ph/Pr DIBH	Ph/Pr SB
	DIBH		SB		p-value DIBH-SB	DIBH		SB		p-value DIBH-SB	p-value	p-value
	Mean	Range	Mean	Range		Mean	Range	Mean	Range			
Heart_mean dose	2.54	(1.43–4.15)	4.55	(2.40–6.58)	< 0.001	0.78	(0.1–2.11)	1.34	(0.53–2.5)	< 0.001	< 0.001	< 0.001
**No cardiac risk factor-no smoking**												
Risk cardiac death (0.075%/Gy (RBE))	0.19	(0.11–0.31)	0.34	(0.18–0.49)		0.06	(0.01–0.16)	0.10	(0.04–0.19)			
Risk cardiac death (1/N)	525	(932–321)	293	(556–203)		1712	(1333–632)	994	(2516–533)			
**Cardiac risk factor(s) or smoking**												
Risk cardiac death (0.3%/Gy (RBE))	0.76	(0.43–1.25)	1.36	(0.72–1.97)		0.23	(0.03–0.63)	0.40	(0.16–0.75)			
Risk cardiac death (1/N)	131	(233–80)	73	(139–51)		428	(3333–158)	249	(629–133)			
Lungs_mean dose	2.71	(1.72–4.45)	3.08	(2.04–4.89)	< 0.001	1.78	(1.05–2.80)	1.44	(0.79–2.34)	< 0.001	< 0.001	< 0.001
**No smoking**												
Risk lung cancer death (0.06%/Gy(RBE))	0.16	(0.10–0.27)	0.18	(0.12–0.29)		0.11	(0.06–0.17)	0.09	(0.05–0.14)			
Risk lung cancer death (1/N)	616	(969–375)	542	(817–341)		936	(1587–595)	1161	(2110–712)			
**Continuing smoking**												
Risk lung cancer death (0.88%/Gy(RBE))	2.38	(1.51–3.92)	2.71	(1.80–4.30)		1.57	(0.92–2.46)	1.26	(0.70–2.06)			
Risk lung cancer death (1/N)	42	(66–26)	37	(56–23)		64	(108–41)	79	(144–49)			
Heart disease*lung cancer mortality: 1 − **∏(1 − p) (%)**												
Low-risk patients (no smoking, no cardiac risk factors) (%)	0.35	(0.21–0.58)	0.52	(0.30–0.79)	< 0.001	0.17	(0.07–0.33)	0.19	(0.09–0.33)	< 0.001	< 0.001	< 0.001
No smoking, cardiac risk factors (%)	0.92	(0.53–1.51)	1.55	(0.84–2.26)	< 0.001	0.34	(0.09–0.80)	0.49	(0.21–0.89)	< 0.001	< 0.001	< 0.001
Smoking, no cardiac risk factors (%)	2.57	(1.62–4.22)	3.04	(1.97–4.78)	< 0.001	1.63	(0.93–2.62)	1.36	(0.73–2.24)	< 0.001	< 0.001	< 0.001
High-risk patients (smoking, cardiac risk factors) (%)	3.12	(1.94–5.11)	4.03	(2.50–6.19)	< 0.001	1.80	(0.95–3.08)	1.66	(0.85–2.79)	< 0.001	< 0.001	< 0.001

Risk estimations for radiation-induced mortality, based on Taylor et al.^7^. Over a 30-year period for a 50-year old (reference) patient, the absolute risk of radiation-induced cardiac mortality was estimated 0.075%/Gy and 0.3%/Gy mean heart dose for patients without and with cardiac risk factors, respectively. For radiation-induced lung cancer mortality, the risk was estimated 0.06%/Gy and 0.88%/Gy mean lung dose (both lungs) for patients who never smoked or continued smoking since adolescence, respectively. The rows showing risk cardiac or lung cancer death (1/N) give the values of N where 1 out of N reference patients treated would die from radiation-induced cardiac injury or lung cancer, respectively, during a 30-year follow-up period. The heart disease*lung cancer mortality is the cumulative 30-year risk in (reference) patients who have cardiac risk factors and/or continue smoking. Mortality risks can be compared to the disease-specific survival benefit of adjuvant WBI + LNI including MI, which we assumed to be ≥ 8% at 30 years^23^. Legend: The columns ‘p-value DIBH-SB’, ‘p-value Ph/Pr DIBH’ and ‘p-value Ph/Pr SB’ represent the p-values of a t-test between the results of a comparison between (respectively): DIBH and SB, photon and proton for DIBH, and photon and proton for SB.

**Figure 4:**
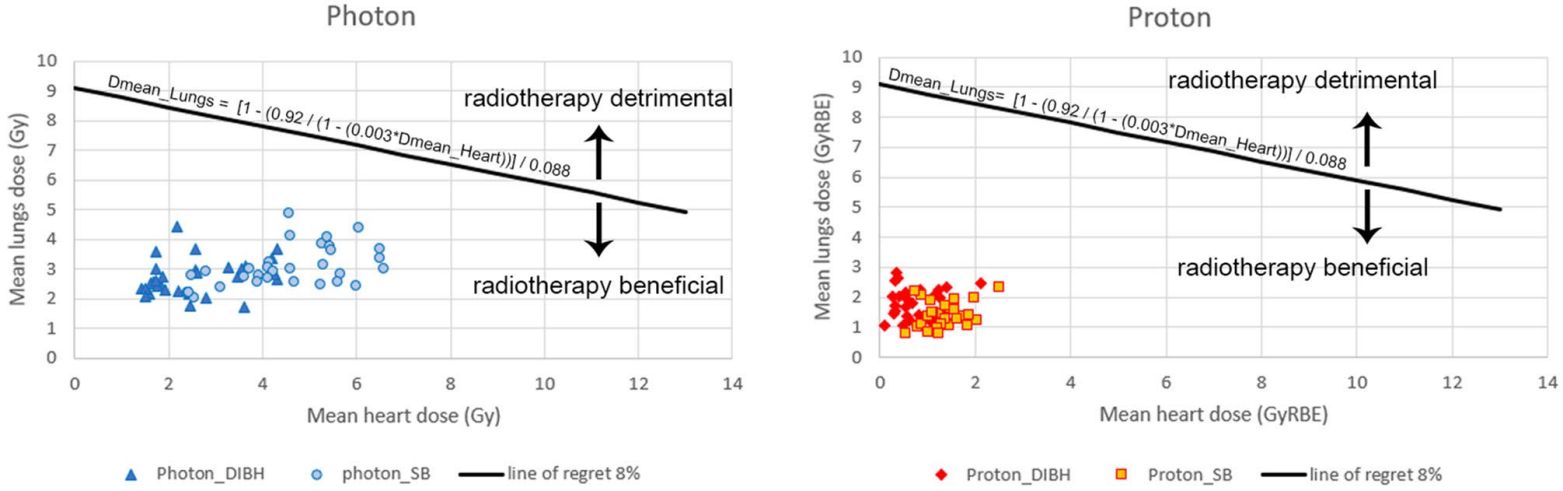
Risk–benefit classification of high-risk patients for cardiac events and lung cancer. Risk calculations based on cardiac and lung cancer mortality neglect risk-contributions from other radiation-induced cancers, such as esophageal, thyroid or contralateral breast cancer. Hence, radiation-related mortality risk is underestimated. Taylor’s data^7^ are based on a variety of prescription doses, the most common being 25 × 2.0 Gy. The prescription dose in this study was 15 × 2.67 Gy. A weakness of these risk calculations is that neither total dose nor fractionation could be taken into account.

Figure 4 shows a risk–benefit classification of high-risk patients of both cardiac events and lung cancer. For none of the patients in this study, the 8% disease-specific survival benefit^23^ from radiotherapy would be outweighed by radiation-induced cardiac or lung cancer mortality. Data points on the line of regret show where the 8% disease-specific survival benefit of radiotherapy is compensated by the survival loss from combined radiotherapy-related cardiac and lung cancer mortality.

## Discussion

DIBH and prone radiation techniques offer significant dose reductions to heart and lung for breast-only irradiation^10,24^. In a factorial design study of prone versus supine and DIBH versus SB for WBI, the combination of prone positioning and DIBH was shown to achieve lower heart and lung doses than any other combination^10^. Patients requiring both breast and regional lymph node (locoregional) irradiation receive even higher lung and heart doses, but at present technical reasons hamper the combination of DIBH and prone radiotherapy and this combination cannot be delivered for routine care. Therefore, we adapted our prone DIBH-technique for use on the Prone Crawl Breast Couch and showed the potential of the combination for heart and lung dose reductions for irradiation of breast and regional lymph nodes. The relative dose reductions by prone DIBH shown in this study, on average 44% mean heart dose reduction by DIBH, are comparable with those previously obtained for local radiation by Mulliez (i.e. 45% mean heart dose reduction by DIBH in supine; 41% mean heart dose reduction in prone)^25^. Saini reported 48% mean heart dose reduction by DIBH in supine and 9% mean heart dose reduction in prone for local irradiation^10^. These relative reduction rates establish the role of DIBH in photon therapy and support the hypothesis that also for locoregional treatment, the combination of prone positioning and DIBH will allow for achieving substantially lower heart and lung doses than the 3 other combinations of techniques: supine-SB, supine-DIBH and prone-SB.

In the photon SB plans of this study, the mean heart dose was 4.55 Gy (range: 2.4–6.58). This is about double the mean heart dose of 2.54 Gy (range: 1.43–4.31) in the photon DIBH plans. These findings are about the half of the 8.7 Gy mean heart dose reported for left-side prone tomotherapy plans by Kainz^26^. Taylor et al. reported estimations of mean heart doses around 8 Gy from locoregional photon irradiation including the MI-chain without breathing control^27^. For photons we thus find that non-coplanar VMAT in the prone crawl position allows to decrease mean heart dose to lower levels than could be achieved by any other photon technique tested by ourselves or known to us from scientific publications. Further decrease of mean heart dose is possible by combining prone positioning with DIBH.

Looking at the individual mean heart doses for photon plans in this study, we find lower heart doses in DIBH for all patients except one. This is due to the creation of a hotspot in the shoulder, cranially outside the lymph node regions. This hotspot derives from the para-sagittal beam direction, as a consequence of trying to spare the heart from dose by lateral beam directions, by which the optimizer was struggling during optimization. In trying to get rid of the hotspot during optimization, the dose to heart increased by enlarging the beam aperture of the lateral beam directions and their dose weights.

Using DIBH in prone crawl position we achieved a mean heart dose of 0.78 GyRBE (range: 0.1–2.11) using protons. For proton prone crawl position during SB the mean heart dose was 1.34 GyRBE (0.53–2.5). Taylor et al. reported estimations of mean heart doses from locoregional irradiation including the MI-chain using protons^27^ around 2.5 GyRBE. Hence, our results correspond well with previously reported results.

**Figure 5:**
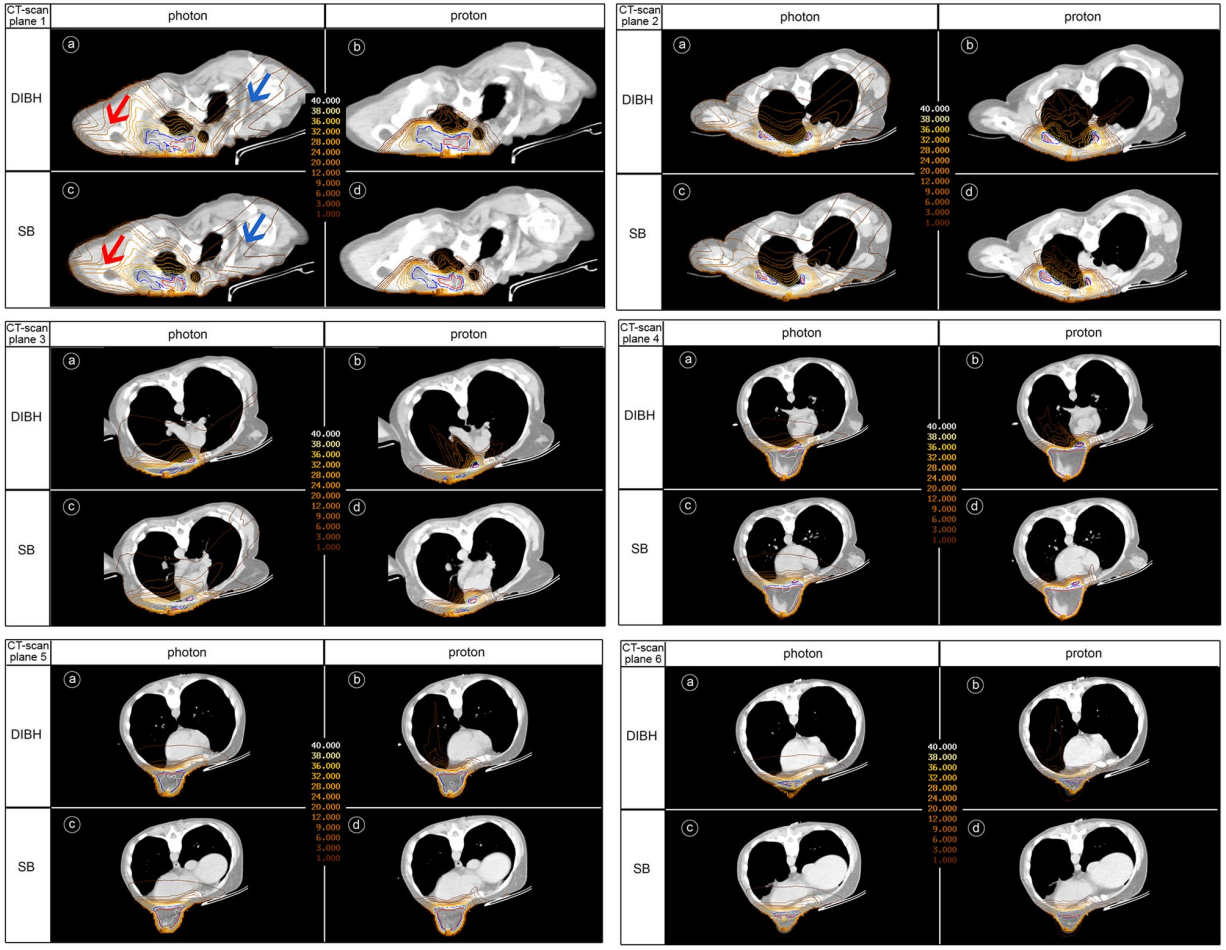
Transverse dose distribution differences between DIBH and SB photon and proton plans. For photon VMAT DIBH and SB (CT scan planes 1–6: **a,c)** plans, a large dose spread is seen outside the target volumes in the dorsal shoulder region and inside the upper thorax^13^. For IMPT proton plans, a larger dose spread is clearly observed in DIBH where tissues of high density were replaced by tissues of low density (CT scan planes 1–6: **b,d**). Plane 1: lung top. Plane 2: near the cranial edge of the left internal mammary lymph node chain. Plane 3: near the cranial edge of the left breast. Plane 4: near the caudal edge of the internal mammary lymph node chain at the central part of the left breast. Plane 5: through the caudal quadrants of the left breast. Plane 6: near the caudal edge of the left breast. CT scan planes 3–6 show in the SB setting **(d)** that distal edges of proton pencils are located in the heart, blood vessels and pericardial fat. These ‘high’ density tissues protect the lung in SB. In the DIBH proton setting **(b)**, high density tissue is replaced by low density lung tissue resulting in increased proton range. Proton dose is deposited far outside the deep edge of the target into the left lung tissue in the DIBH plan **(b)** compared to SB plan **(d)**.

The dose spread difference between the photon and proton modality in this study is clarified in Fig. 5. In the intra-thoracic and dorsal shoulder region, ratios of larger dose spread were found for photon (both DIBH and SB) than for proton plans, similar to our previous study^13^. The main difference in this study is the mutual ratio of dose spread in lung between DIBH and SB photon and proton modality, respectively. In the DIBH proton setting, we found higher mean lung doses whereby a larger dose spread is seen in low density cavities. This can be explained by anatomical differences between SB and DIBH. In SB, the heart occupies a larger part of the left anterior thoracic cavity than in DIBH and displaces lung tissue in dorsal direction out of reach of the proton beams. In SB, the heart functions as a “dose absorber” in front of the lung while in DIBH while in DIBH, the “dose absorber” is retracted medially and caudally leaving space to lung tissue to move closer to the irradiated breast where it can be reached by the proton beams. The lower mean lung dose in DIBH photon plans, compared to SB photon plans, can be explained by the use of non-coplanar VMAT beam directions and the gain of unexposed lung volume expansion in the posterior and caudal direction by DIBH .

The use of multiple short breath holds of 15–30 s represents the most common mode of implementation of DIBH. Clinical experience indicates that multiple short breath-holds are more challenging with the addition of regional irradiation. Whereas a local radiotherapy session is typically completed using 3–6 multiple short breath-holds each of 12–18 s and most patients can be trained to perform these easily, we find that a locoregional radiotherapy session requires 10–14 multiple short breath-holds each of 15–30 s. To our experience, this represents a substantial physical and mental effort for all but the most able patients . At the University and Queen Elizabeth Hospital (Birmingham, UK), a single prolonged breath-hold technique was developed, using pre-oxygenation and asymptomatic hypocapnia induced by mechanical hyperventilation^28–32^. Volunteers and breast cancer patients were able to maintain safely and comfortably single prolonged breath-holds of 5 min and more. Another solution could be the use of percussive ventilation^33^. An entire locoregional radiotherapy session could therefore be delivered in theory in one single prolonged breath-hold. The prolonged breath-hold technique is presently being translated for use in the prone crawl position.

Using modern radiation techniques such as IMPT and PBS, proton therapy is able to decrease OAR-dose beyond what is possible with photon techniques^13^. However, breathing motion may jeopardize the accuracy of proton therapy because it may induce uncertainty in proton range and dose prediction. Motion of breast, shoulder and sternal regions by breathing is smaller in prone crawl than in supine position and dose prediction uncertainty is also expected to be smaller^34^. DIBH reduced mean heart, apex and LAD doses on average by 42% (0.78–1.34 GyRBE), 21% (3.44–4.35 GyRBE) and 3% (2.19–2.26 GyRBE) respectively, but increased left and right lung mean doses by 21% (3.70–3.05 GyRBE) and 75% (0.14–0.08 GyRBE), respectively.The clinical impact is highly dependent on patient-individual risk factors, shown in the regret-analysis of Fig. 4. The relative increase in dose for both lungs combined was 46%. Epidemiological analyses of the Oxford group suggest that the risk of lung cancer induction is linear with the lung mean dose with no threshold^6,7^. These investigators fitted the epidemiological data to the mean dose of both lungs combined. At present, we lack the parameters to calculate risk of lung cancer separately for each lung. The use of couch rotation or novel techniques like proton arc therapy and real-time motion monitoring in respiratory-gated PBS proton therapy could mitigate this issue^35^. DIBH resulted further in small dose increases in esophagus, contralateral (right) breast and spinal cord. The cause of opposite dose changes on heart and lung is explained above. Using DIBH in prone locoregional breast proton therapy can be beneficial, neutral or detrimental depending on the clinical situation. In patients with cardiac but no lung cancer risk factors, overall risk reduction can be expected from DIBH. In patients with lung cancer but no cardiac risk factors, SB would be indicated, and end-expiratory breath hold might be worth investigating.

This publication is the first that reports quantitative results of prone DIBH for locoregional irradiation of breast cancer. Data on direct comparison of prone DIBH and supine DIBH are inexistant for this patient group. For irradiation of the breast only, the 2 existing publications^10,25^ show that the lowest mean heart and lung doses are achieved for prone DIBH.

During the past decades, gating technology has been developed for photon and proton radiotherapy. Respiratory gating requires that the radiation beam starts with minimal delay after the intended breath hold depth is reached and is paused immediately after a pre-set length of breath hold or when the breath hold depth is out of a pre-set range. Long or unpredictable delays to restart the beam after pausing was a major hurdle to implement multiple DIBH in the past. This problem is resolved with modern technology and multiple DIBHs can be clinically implemented in photon or proton radiotherapy. Errors in DIBH may result in geometrical errors of target and OAR shape and position. Geometrical errors generally translate to larger dose errors in proton than in photon therapy. Prone DIBH can be expected to be more robust than supine DIBH for photon as well as for proton therapy because respiration-related motion of the target volume is smaller in prone than in supine position^34^, but this needs further investigation.

## Conclusion

We investigated the potential benefits of prone crawl positioning in WB + LN (including MI) RT by evaluating the dosimetrical effects of DIBH and SB in photon and proton plans. DIBH significantly decreased doses to heart for proton and photon radiotherapy. For photons, the relative reduction establish the role of DIBH in photon RT. This supports the hypothesis that also for locoregional treatment, the combination of prone positioning and DIBH will allow for achieving substantially lower heart and lung doses than the 3 other combinations of techniques: supine-SB, supine-DIBH and prone-SB. For DIBH in proton plans, an increase of lung dose should be taken into account. The radiation-related mortality risk could not outweigh the ~ 8% disease-specific survival benefit of WB + LN_MI radiotherapy in any of the assessed treatments.
