# Author Correction: Effects of deep inspiration breath hold on prone photon or proton irradiation of breast and regional lymph nodes

**DOI:** 10.1038/s41598-023-40643-2

**Published:** 2023-08-23

**Authors:** Bruno Speleers, Max Schoepen, Francesca Belosi, Vincent Vakaet, Wilfried De Neve, Pieter Deseyne, Leen Paelinck, Tom Vercauteren, Michael J. Parkes, Tony Lomax, Annick Van Greveling, Alessandra Bolsi, Damien C. Weber, Liv Veldeman, Werner De Gersem

**Affiliations:** 1 Department of Human Structure and Repair, Faculty of Medicine and Health Sciences, Ghent University, Radiotherapiepark, Corneel Heymanslaan 10, 9000 Ghent, Belgium; 2 Paul Scherrer Institut, Villigen, Switzerland; 3 Department of Radiation Oncology, University Hospital Ghent, Ghent, Belgium; 4 Department of Industrial Systems Engineering and Product Design, Faculty of Engineering and Architecture, Ghent University, Ghent, Belgium; 5 Academic Medical Centre (AMC), University of Amsterdam, Amsterdam, the Netherlands; 6 Radiation Oncology Department, University Hospital of Bern, Bern, Switzerland; 7 Radiation Oncology Department, University Hospital of Zurich, Zurich, Switzerland

Correction to: *Scientific Reports* 10.1038/s41598-021-85401-4, published online 16 March 2021

The Acknowledgements section in the original version of this Article was incomplete. It now reads:

“This work was financed through grant FAF-C/2018/1190 of the Foundation against Cancer. Annick Van Greveling has been funded by a grant of Think-Pink. Liv Veldeman is recipient of post-doctoral clinical mandate of Foundation against Cancer. Prototype research is funded by StarTT 241 grant of the Industrial Research Fund, Ghent University). Dr Parkes was funded by a Marie Sklodowska-Curie Individual Fellowship “Mechanical ventilation for radiotherapy” (894619) from the European Commission.”

The original Article has been corrected.

